# Effects of pomegranate juice (*Punica Granatum*) on inflammatory biomarkers and complete blood count in patients with COVID-19: a structured summary of a study protocol for a randomized clinical trial

**DOI:** 10.1186/s13063-021-05194-9

**Published:** 2021-04-02

**Authors:** Mojtaba Yousefi, Mohammadreza Sadriirani, Azizollah PourMahmoudi, Sara Mahmoodi, Bahar Samimi, Mahboobe Hosseinikia, Zaker Saeedinezhad, Seyed Bahman Panahande

**Affiliations:** 1 Department of Nutrition, School of Health and nutrition, Yasuj University of Medical Sciences, Yasuj, Iran; 2 Department of Internal Medicine, School of Medicine, Yasuj University of Medical Sciences, Yasuj, Iran

**Keywords:** COVID-19, Randomized Controlled Trial, Protocol, Pomegranate Juice, CRP, IL-6, CBC, ESR

## Abstract

**Objectives:**

This study is conducted to investigate efficacy of pomegranate juice on inflammatory biomarkers, C-reactive protein (CRP), interleukin 6(IL-6), erythrocyte sedimentation rate (ESR) and complete blood count (CBC) in hospitalized patients with mild to moderate coronavirus disease 2019 (COVID- 19).

**Trial Design:**

This is a randomized, placebo-controlled, double-blind, parallel 2-arm (1:1 ratio) clinical trial.

**Participants:**

Patients with COVID-19 admitted to hospitals in Yasuj City, Kohgiluyeh and Boyer-Ahmad Province, Iran.

**Inclusion Criteria:**

- Informed consent
- Patients 18 years of age or older
- Diagnosis of COVID-19 based on real-time polymerase chain reaction (RT-PCR) test

**Exclusion Criteria:**

- Pregnancy or lactation
- Immunoglobulin A (IgA) level <61 mg/dl
- Disseminated intravascular coagulation or any other types of coagulopathy
- Severe congestive heart failure
- Participation in any clinical trial within 30 days prior to enrollment in this RCT
- Other contraindications determined by the specialist.

**Intervention and Comparator:**

Intervention: 500 ml pomegranate juice and standard of care hospital treatment for COVID-19

Comparator: matching placebo containing 500 ml of red water and standard of care hospital treatment for COVID-19

Both intervention and comparator to be taken twice a day, after lunch and dinner, for 14 days.

**Criteria for Discontinuing:**

Transfer of patients to intensive care unit (ICU)

Death

Unwillingness to continue participating in the study

**Main Outcomes:**

The main outcomes of this study are levels of inflammatory biomarkers, CRP, IL-6, ESR, and CBC after 14 days of treatment.

**Randomization:**

Eligible patients will be randomly assigned into the intervention or control group in a 1:1 ratio. Randomization will be performed based on 8 permuted blocks with block sizes of 6 and they will be stratified according to sex and age categories. Randomization sequences will be prepared by the trial’s pharmacist using computer-generated random numbers.

**Blinding (Masking):**

This study is a double-blind clinical trial (participant, researcher). The pomegranate juice and placebo juice are packaged in identical bottles, and the researcher and all the patients will be unaware of the study assignment until the end of the study. To ensure blinding, the randomization sequences will be kept in identical, opaque, sealed, and sequentially numbered envelopes.

**Numbers to Be Randomized (Sample Size):**

The calculated total sample size is 48 patients, with 24 patients assigned into each group.

**Trial Status:**

The protocol is Version 1.0, on March 3, 2021. Recruitment started on February 28, 2021, and is anticipated to be completed by May 21, 2021.

**Trial Registration:**

The Name of registering trial

Effects of Pomegranate Juice (*Punica Granatum*) on Inflammatory Biomarkers and CBC in Patients with COVID-19: A Randomized, Double-Blind, Placebo-Controlled Clinical Trial.

Iranian registry of clinical trials (IRCT) Registration Number: IRCT20150711023153N2

Date of Trial Registration

February 28, 2021, retrospectively registered

**Full Protocol:**

The full protocol is attached as an additional file, accessible from the Trials҆ website (Additional file 1). In the interest of expediting dissemination of this material, the familiar formatting has been eliminated; this Letter serves as a summary of the key elements of the full protocol.

**Supplementary Information:**

The online version contains supplementary material available at 10.1186/s13063-021-05194-9.

## Supplementary Information

**Additional file 1.**

## Data Availability

The final dataset of the trial will be available upon request from the primary investigator via e-mail at panahande.b@gmail.com, after obtaining permission from the Regional Ethics Committee.

