# Supplementary material for: Effects of pomegranate juice (Punica Granatum) on inflammatory biomarkers and complete blood count in patients with COVID-19: a structured summary of a study protocol for a randomized clinical trial
Source: Trials. 2021 Apr 2;22:246. doi: 10.1186/s13063-021-05194-9 (PMC8017515; doi:10.1186/s13063-021-05194-9)
Supplement: Supplementary file 1 — Additional file 1. [file 13063_2021_5194_MOESM1_ESM.pdf]

Effects of Pomegranate juice (*Punica granatum*) on inflammatory biomarkers and CBC in patients with covid-19: Study protocol for a randomized clinical trial

Backgrounds and Objectives:

Coronaviruses are a large family of viruses and a subset of coronaviridae that have been seven generations of this family have been discovered [1, 2]. This virus family was first introduced in 1965 which is most spread in mammals [2]. SARS-CoV2 is the last generation of this virus family, which was first discovered in 2019 in Wuhan, China. [3] This virus is classified as a Positive-strand RNA virus [2]. Most prevalent symptoms of this infection include headache, fever, dry cough, fatigue, maldigestion, and shortness of breath [4]. The incubation period varies from 2 to 14 days depending on the underlying diseases such as diabetes, hypertension, chronic obstructive pulmonary disease, cardiovascular and kidney disease, and the immune system function [5]. The severity of symptoms is individualized and varies from mild to inflammatory and fatal conditions. The mild type of this disease is known as the absence of pneumonia, but severe type respiratory, hypoxia and organ failure are more pronounced in severe and fatal types. Organ function or organ failure is seen [1]. The main transmission rout of covid-19 is thorough respiratory droplets and close contact. RT-PCR is the most accurate diagnostic test, but CT scan and blood test can also be used [2, 5, 7, 8]. Previous studies have demonstrated that a number of people with the severe type of Covid-19 display symptoms of cytokine syndrome. This disorder which is often caused by overactive immune system, is a hyper inflammatory syndrome known as sudden and fatal hypercytokinemia and multiple organ failure (MOF) [9, 10]. The main mechanism of pathogenesis is the binding of the virus to angiotensin 2 receptors, which increases the activity of T lymphocytes and thus increases the production of inflammatory factors [1]. According to the above-mentioned statement, it seems that people whom boost their immune system, with the help of drugs, supplements and foods have been able to prevent the progression of inflammatory processes and overcome the disease [1, 2]. Therefore, immune boosting foods such as pomegranate could be considered as a potential therapeutic option [1-3, 11]. Pomegranate is a fruit with numerous antiviral and antioxidant compounds, including: polyphenols, flavonoids, tannins, anthocyanins and flavonoids, vitamins E and C, magnesium, zinc, potassium, iron and quercetin, whose beneficial effects on the various diseases including cardiovascular diseases and diabetes have been studied and have an enormous capacity to neutralize free radicals mainly because of ellagic acid and pony collagen [12-16] [16-19]. As reported by Feng et al. these polyphenols have an inhibitory effect on NF-KB pathway which has a pivotal role in disease pathogenesis [18]. Reducing CRP and inhibiting the cytokine storm

pathway through the inhibitory effects of conjugated terpenes on PPAR factors is the other protective mechanisms [15, 20]. As far as our knowledge goes, this work is the first study which examines the anti-inflammatory effects of whole pomegranate juice on disease progression in covid-19 patients.

Material and Methods:

Participants & recruitment:

Out of the total number of hospitalized patients, 48 patients with definitive diagnosis based on PCR test, who also have other inclusion criteria, are included in the study by availability sampling.

Study Design & randomization:

The present study will be performed as a Randomized, Placebo-Controlled, Double-Blind, Parallel-Group clinical trial on patients with Covid-19 admitted to Shahid Jalil Hospital in Yasuj (Kohgiluyeh & Boyr Ahmad province, south-west of Iran). The study protocol was approved by the Ethics Committee of the School of Nutrition and Public Health () in Yasuj University of Medical Sciences and is in agreement with the declaration of Helsinki of the world medical association. This study was also registered in the Iranian Center for Clinical Trials (). Patients will be randomly divided into two groups of intervention (n=24), and placebo (n=24), by block random sampling method, which randomizes participants into 4 different blocks in each group, based on gender and age . Intervention group receive 500 ml of natural whole pomegranate juice twice a day, after lunch and dinner and control group receive the same amount of placebo with the same color and taste as natural juice. The pomegranate juice and placebo will be administered to patients by two nurses in opposite shifts. Patients will be followed for 14 days and the objectives of the study will be fully explained to the patient and their companions. An informed written consent form will be also obtained.

Preparation of pomegranate juice:

Pomegranates of Iranian species (robab) will be picked by hand and stored in tanks. All fruits will be crushed with skin. The juice will be pasteurized, concentrated and filtered at a

temperature of 18 ° C. The product will be packaged in a disposable bottle and given to the patient daily. According to the manufacturer's recommendation, pomegranate juice will be stored at room temperature (25 ° C) until consumption [17].

Preparation of placebo drink:

Placebo is prepared by the addition of 0.02 % pomegranate emulsion of Mongolia Company to water. This protocol is approved by the Ministry of Health and is in accordance with the instructions on the product. The color is also matched to the natural pomegranate juice by edible color with the international code of E-122 [21].

Study eligibility:

Inclusion criteria:

- written informed consent form
- Age 18 years old or above
- COVID-19 diagnosis based on polymerase chain reaction (RT-PCR)
- Acceptance for random assignment
- Willingness to participate in research study

Non-inclusion criteria

- Pregnancy or lactation
- IgA <61 mg/dl
- Disseminated intravascular coagulation or any other types of coagulopathy
- Severe congestive heart failure
- Participate in any clinical trial within the past 30 days prior to enrollment in the present work
- Other contraindications prescribed by the specialist.

Exclusion criteria

- Transfer of patients to the intensive care unit

- Death
- Unwillingness to continue participating in the study

Clinical, Para clinical and dietary intake assessments:

A demographic questionnaire containing information about age, sex, habitat, education, occupation etc., will be taken from patients. Medical history and medications/supplements history will also be asked of the patient or his companion.

Height, weight and waist to hip ratio (WHR) will be measured by standard methods [1] as well as the body mass index (division of weight in kilograms to the height in meters squared).

For the purpose of evaluating detailed information about nutrient intake and the food pattern, a 24-hour dietary recall for 3 days (including a weekend day) will be taken by a well-trained interviewer. Dietary intakes will be entered into Nutritionist IV software for further analysis.

10 cc blood samples will be obtained after 12 hour overnight fasting to measure complete blood count (CBC) and inflammatory indices including CRP, ESR & IL-6, at the baseline and by the end of week-2. In the case of patient discharge earlier than scheduled, they will be followed up after discharge and receive pomegranate juice or placebo. The length of hospital stay, disease complications, drug and prescription dosing and mortality rate will be recorded on a daily basis. If the patient refuses to continue the study for any reason, blood sample will be drawn on the same day.

Follow up:

Subsequent follow-up is to detect and minimize the effects of the confounding factors, as well as to accurately carry out the intervention protocol and will be done by well-trained, committed researchers.

Study objectives:

Primary objectives

To assess of the effect of Punica granatum juice on inflammatory biomarkers and CBC in patients with Covid-19

Secondary objectives

- To assess and compare the mean or median serum concentrations of interleukin 6 before and after the intervention and between the intervention group with placebo
- To assess and compare the mean or median serum concentrations of ESR before and after the intervention and between the intervention group with placebo
- To assess and compare the mean or median serum concentrations of CRP before and after the intervention and between the intervention group with placebo
- To assess and compare the mean or median of CBC parameters before and after the intervention and between the intervention group with placebo

Data analysis

The data will be analyzed by SPSS software version 25. After examining the normality distribution of variables using Kolmogorov-Smirnov test, quantitative normal variables will be reported as mean±standard deviation, quantitative non-normal variables will be reported as median IQR, and qualitative variables as number (percentage). Independent T Test and Mann Whitney test will be used to compare the mean of variables between two groups. Paired T test or Wilcoxon test will be used to analyses within groups means. P-Value <0.05 will be considered as statistically significant. If the patient refuses to continue the study for any reason, statistical methods (Intention-to-treat analysis) will be performed.

1. STEPS, W., *Section 3: guide to physical measurements (Step 2)*, 2008, Geneva.

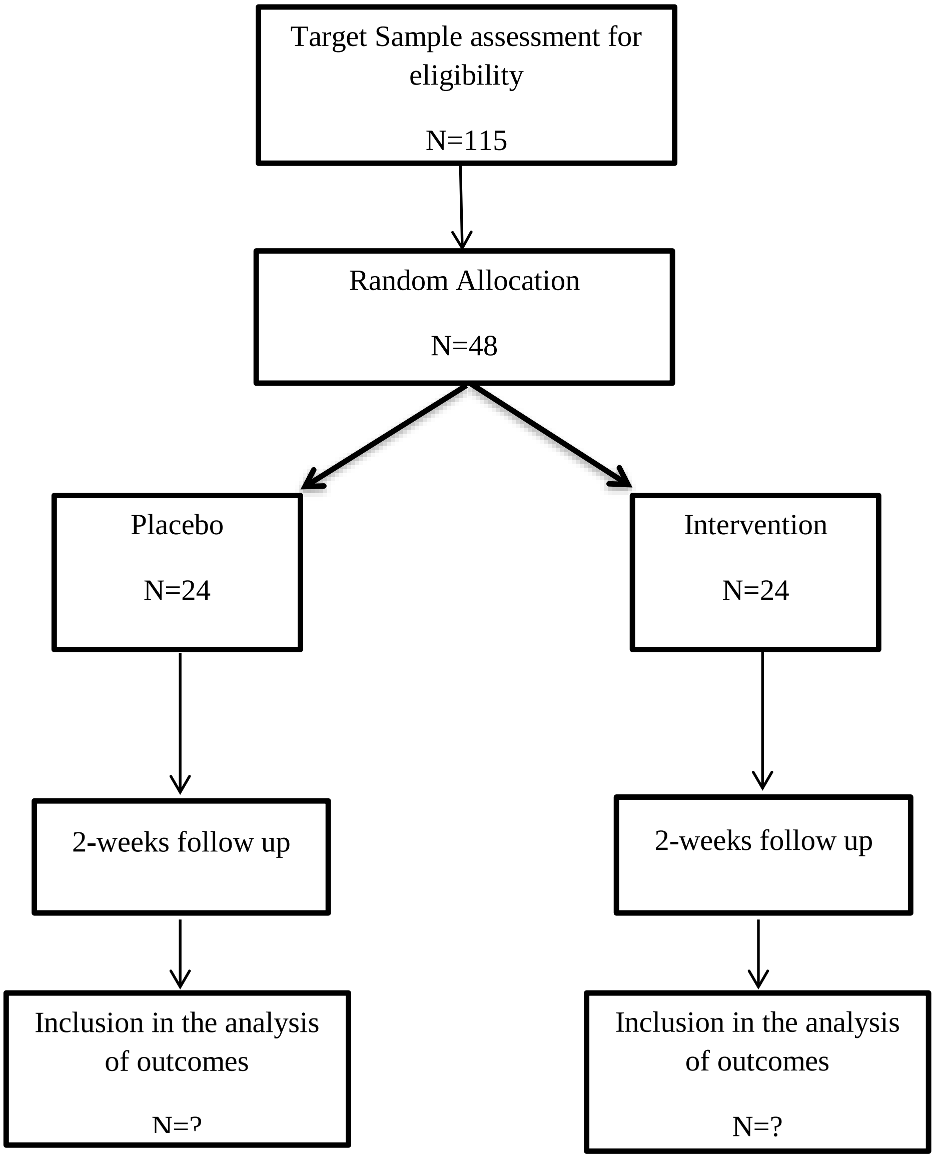

Figure 1: Study Process Flow chart
